# Transcription Profiling of Monocyte-Derived Macrophages Infected *In Vitro* With Two Strains of *Streptococcus agalactiae* Reveals Candidate Pathways Affecting Subclinical Mastitis in Cattle

**DOI:** 10.3389/fgene.2019.00689

**Published:** 2019-07-26

**Authors:** Anna Monika Lewandowska-Sabat, Elena Kirsanova, Christophe Klopp, Trygve Roger Solberg, Bjørg Heringstad, Olav Østerås, Preben Boysen, Ingrid Olsaker

**Affiliations:** ^1^Department of Basic Sciences and Aquatic Medicine, Faculty of Veterinary Medicine, Norwegian University of Life Sciences, Oslo, Norway; ^2^Sigenae, UBIA INRA, Castanet-Tolosan, France; ^3^Geno Breeding and A.I. Association, Hamar, Norway; ^4^Department of Animal and Aquacultural Sciences, Faculty of Biosciences, Norwegian University of Life Sciences, Ås, Norway; ^5^Norwegian Cattle Health Services and TINE Extension Services, Ås, Norway; ^6^Department of Food Safety and Infection Biology, Faculty of Veterinary Medicine, Norwegian University of Life Sciences, Oslo, Norway

**Keywords:** cattle, macrophages, pathway analysis, RNA sequencing, *Streptococcus agalactiae*, subclinical mastitis

## Abstract

Macrophages are key cells of innate immune response and serve as the first line of defense against bacteria. Transcription profiling of bacteria-infected macrophages could provide important insights on the pathogenicity and host defense mechanisms during infection. We have examined transcription profiles of bovine monocyte-derived macrophages (bMDMs) isolated from the blood of 12 animals and infected* in vitro* with two strains of *Streptococcus agalactiae*. Illumina sequencing of RNA from 36 bMDMs cultures exposed *in vitro* to either one of two sequence types of *S. agalactiae* (ST103 or ST12) for 6 h and unchallenged controls was performed. Analyses of over 1,656 million high-quality paired-end sequence reads revealed 5,936 and 6,443 differentially expressed genes (*p* < 0.05) in bMDMs infected with ST103 and ST12, respectively, versus unchallenged controls. Moreover, 588 genes differentially expressed between bMDMs infected with ST103 versus ST12 were identified. Ingenuity pathway analysis of the differentially up-regulated genes in the bMDMs infected with ST103 revealed significant enrichment for granulocyte adhesion and diapedesis, while significant enrichment for the phagosome formation pathway was found among down-regulated genes. Moreover, Ingenuity pathway analysis of the differentially up-regulated genes in the bMDMs infected with ST12 showed significant enrichment for type 1/type 2 T helper cell activation, while the complement activation pathway was overrepresented in the down-regulated genes. Our study identified pathogen-induced regulation of key genes and pathways involved in the immune response of macrophages against infection but also likely involved in bacterial evasion of the host immune system. These results may contribute to better understanding of the mechanisms underlying subclinical infection such as bovine streptococcal mastitis.

## Introduction

Monocytes and macrophages are critical cells associated with innate immunity, regulation of inflammation, and host defense against invading pathogens. Macrophages are capable of phagocytosis, a major immune mechanism used to remove pathogens, and local recruitment and action of macrophages in the mammary gland is an essential immunological defense mechanism against infection such as mastitis (Politis et al., 1991; Barber and Yang, 1998; Riollet et al., 2001; Alluwaimi et al., 2003). Intramammary infections will trigger macrophages to produce not only pro-inflammatory cytokines required to eliminate pathogens but also anti-inflammatory factors essential for immune regulation of the inflammation and preventing chronic conditions (Gunther et al., 2016).

Mastitis is a multi-factorial disease, affected by load and virulence of infecting pathogens and host genotype. Some cows develop chronic subclinical mastitis with high somatic cell count (SCC) in milk. Such animals may shed the bacteria and contribute to infection spreading to other cows and herds. However, the mechanisms that underlie the pathogenesis of subclinical mastitis are not well understood.

In the last decade, genomic selection has been implemented in animal breeding worldwide. Large genetic differences exist in cows’ susceptibility to mastitis (Heringstad et al., 2007). Routine genetic evaluation of udder health of Norwegian Red is based on information both on veterinary treatment of clinical mastitis and on SCC. SCC is an indicator of mastitis widely used in genetic evaluation of udder health (Miglior et al., 2017). Genomic breeding values (GEBV) for SCC can be used to identify cows with different susceptibility to chronic subclinical mastitis.

The first objective of this study was to examine the genome-wide transcription profiles of primary bovine monocyte-derived macrophages (bMDMs) infected *in vitro* with two *Streptococcus agalactiae* strains, one of the leading causative agents of subclinical bovine mastitis worldwide. Strain ST103 is the most prevalent bovine *S. agalactiae* isolate found in farms with considerable environmental contamination, while ST12, a strain usually associated with colonization of pregnant women, has been found in cattle herds with no positive environmental samples (Manning et al., 2009; Jorgensen et al., 2016). The second objective was to examine differences in transcript profiles of bMDMs isolated from two groups of animals differing in their genomic (low vs high GEBV for SCC) and phenotypic (high vs low SCC) characteristics.

To the best of our knowledge, this study is the first report of genome-wide transcriptome profiling of bMDMs infected with *S. agalactiae*. The identification of key differentially expressed genes and pathways in infected bMDMs provides a basis for improved understanding of central mechanisms of host defense during subclinical infections such as mastitis.

## Material and Methods

### Animals and Cell Isolation

Twelve healthy Norwegian Red cows aged 4–8 years were used in the experiment. The cows came from three Norwegian herds, and both animals with low (total *n* = 6) and high (total *n* = 6) GEBV were selected from each farm to avoid potential farm induced bias. Among these 12 animals, three with high and nine with low SCC phenotype were found (for details, see **Supplementary Table 1**).

Five hundred milliliters of blood was collected from the neck of each animal (*n* = 12) in sterile glass bottles with sodium citrate as anticoagulant. Peripheral blood mononuclear cells were extracted as described earlier (Lewandowska-Sabat et al., 2013). Briefly, density gradient centrifugation (2,210 × g, 30 min) on lymphoprep (Axis-Shield, Norway) was used, and CD14+ cells were isolated by positive selection of monocyte differentiation antigen CD14 using anti-human CD14 MACS MicroBeads (Miltenyi Biotec GmbH, Bergisch Gladbach, Germany), according to the manufacturer’s instructions. Purity of selected cells was verified by flow cytometry by staining positively selected cells with PE-conjugated anti-mouse IgG2 (Southern Biotech, Birmingham, AL, USA), analyzing in a Gallios flow cytometer (Beckton Dickinson), and found to be in the range of 95–98%. The CD14+ cells were subsequently grown in six-well dishes at a density of 1.5 × 10^6^ cells per well in RPMI medium supplemented with 10% FCS (Invitrogen, Carlsbad, USA). Cells were left over night at 37°C in an atmosphere with 5% CO_2_. The phenotypic morphology of cells, i.e., differentiation of monocytes into an early-stage adherent macrophage phenotype, was visualized and confirmed by phase contrast microscopy.

### Bacterial Infection

Two previously described *S. agalactiae* strains (ST103 and ST12) derived from Norwegian dairy herds were obtained from The Norwegian Veterinary Institute (Jorgensen et al., 2016). These bovine adapted strains were isolated from milk samples. Bacteria were collected from blood agar plates and grown in “Todd Hewitt broth” (Sigma-Aldrich) until mid-log phase as described previously (Lewandowska-Sabat et al., 2018a). Briefly, growth was measured by optical density at 600 nm. The mid-log phase cultures were aliquoted and frozen at −70°C as 20% glycerol stocks. The final number of colony-forming units (CFU) was determined by serial dilutions and plating on blood agar plates. Bacteria used in this study all came from aliquots of the same batch.

For each individual animal (*n* = 12), the wells with bMDMs were grouped into three classes with an equal number of wells and cells per class as possible, constituting in total 36 different samples. Two classes were infected with either ST103 or ST12 using a multiplicity of infection of 1 (1 bacterium per cell, on average). The third cell class was left uninfected (negative control). After 1 h of exposure, 1% of penicillin/streptomycin (60 pg/ml penicillin and 100 µg/ml streptomycin) were added to prevent growth of the remaining extracellular bacteria. The controls and the infected cells were treated equally. Inhibition of bacterial growth by antibiotics was verified by microscopy. Incubation was continued for five more hours for both bacteria-infected cells and negative controls for a total of 6-h incubation (i.e., 6-h infection in the samples exposed to bacteria). Medium was aspirated, and the cells were collected using a cell scraper. Cells were centrifuged (400 × g, 5 min), and the pellet was washed with cold PBS buffer, snap frozen in liquid nitrogen, and stored at −70°C.

### RNA Extraction

Thirty-six different samples of cells, i.e., three classes (control, ST103-infected, and ST12-infected), from each of the 12 animals, were used to extract RNA. Total RNA was isolated from control and infected cells using the MirVANA isolation kit (Ambion, Austin, TX) following the manufacturer’s instructions. All RNA samples were treated with amplification grade DNase I (Invitrogen) to remove any traces of genomic DNA. RNA concentration and quality was measured using NanoDrop 1000 (Thermo Fisher Scientific, Wilmington, USA) and 2100 BioAnalyzer with Agilent RNA 6000 Nano kit (Agilent Technologies, Palo Alto, USA), respectively. The RNA integrity numbers (RIN), concentrations, and optical density A260/A280 ratios are listed in **Supplementary Table 2**.

### RNA Sequencing and Data Analyses

The 36 RNA samples (control = 12, ST12 = 12, ST103 = 12; **Supplementary Table 3**) extracted above were subsequently submitted for RNA deep sequencing. RNA-seq libraries were prepared from 500 ng of total RNA using TruSeq Stranded mRNA prep kit with polyA enrichment (Illumina), according to the manufacturer’s protocol. Libraries were sequenced (150 bp paired-end, 250–300 M reads per end) on five lanes with an Illumina HiSeq 3000 machine (Illumina). Preparation of RNA-seq libraries and sequencing were performed by the Norwegian Sequencing Centre (Oslo, Norway; http://www.sequencing.uio.no/).

The read quality of the RNA-seq libraries was evaluated using FastQC software ver. 0.11.2 (http://www.bioinformatics.babraham.ac.uk/projects/fastqc/). Cutadapt ver. 1.8.3 (http://www.cutadapt/) was then used to trim adaptor sequences. Reads were mapped against the bovine genome assembly v.3.1. (UMD3.1) using the STAR aligner (v.2.3.1y) (Dobin et al., 2013).

The “rsem prepare reference” script of RSEM package was used to generate reference transcript sequences by using gene annotation file (GTF format) and the full genome sequence (FASTA format) of Bos taurus v.3.1. The calculation of relative transcript abundances in each sample was performed by the “rsem calculate expression” script of the RSEM package (Li and Dewey, 2011).

### Differential Expression Analysis

In order to identify statistically significant differentially expressed (DE) transcripts between negative control and treatment samples, DESeq2 ver. 1.4.5. (Love et al., 2014) and edgeR ver.3.20.9 (Robinson and Oshlack, 2010) were applied in R ver.3.4.1 (http://www.R-project.org). The gene raw counts obtained from RSEM were used as an input, and genes with low expression were removed with a minimal set threshold of one count per million in edgeR. The analyses were performed pairwise, i.e., negative control with one treatment group (ST103 or ST12, respectively). In addition, a comparison between the two bacterial treatment groups (ST103 and ST12) was performed.

Furthermore, a pairwise comparison between low (*n* = 6) and high (*n* = 6) GEBV animals was performed for each treatment (e.g., negative control low GEBV vs negative control high GEBV, ST103 low GEBV vs ST103 high GEBV, etc). We have also performed a pairwise comparison between high (*n* = 3) and low (*n* = 9) SCC animals for each treatment. DE genes were considered significant when both the false discovery rate (FDR) was <0.05 in edgeR and the Benjamini and Hochberg corrected P-value (adjusted) was <0.05 in DESeq2 (i.e., only genes that were identified by both methods as significantly DE are presented in the results and analyzed further). In order to determine how well samples cluster together based on the similarity of their overall RNA expression profiles, hierarchical clustering of the samples was performed in R using “pheatmap” package ver.1.0.8. The heatmap of all samples is presented as **Supplementary Figure 1**.

### Pathway Analysis

Significant DE genes identified both by edgeR and DESeq2, and with a log fold change (logFC) either ≤−1.5 for down-regulated or ≥1.5 for up-regulated as estimated by edgeR were used in constructing Venn diagrams (http://jvenn.toulouse.inra.fr/app/index.html) and for Ingenuity Pathway Analysis (IPA; http://www.ingenuity.com). The list of genes found to be DE between negative controls and ST103- or ST12-infected bMDMs, and between ST103- and ST12-infeceted bMDMs, was used as input in IPA in order to identify canonical pathways, biological functions, and networks overrepresented in the datasets. The down- and up-regulated genes were analyzed separately. Benjamini and Hochberg corrected *P*-value ≤ 0.05 was defined as significant in IPA.

## Results

### Summary Statistics for the RNA-seq Data

For each library, 31–69 million paired-end reads were generated during the sequencing run. Preliminary quality control of the resulting 36 fastq files revealed that all libraries passed the quality criteria with a Phred score > 32. The sequencing resulted in 1,656 million high-quality paired-end reads, and 1,648 million sequences (99.5%) remained after adaptor trimming. A mean of 86.3% of the reads per RNA-Seq sample aligned to unique locations in the Bos taurus v.3.1 genome. The total number of read pairs per sample, the number and percentage of reads passing filters, and the percentage of uniquely mapped reads for each sample are presented in **Supplementary Table 2**. The datasets analyzed during the current study have been deposited at EMBL-EBI (https://www.ebi.ac.uk/ena/) under study accession number PRJEB24166 (Lewandowska-Sabat et al., 2018b).

### Differentially Expressed Genes in Macrophages Challenged With *S. agalactiae*

The difference between the negative control and the ST103- or ST12-infected samples was confirmed by hierarchical clustering (**Supplementary Figure 1**). All control samples clustered together and separately from both the bacterial strain-treated samples.

Analyses of differential expression revealed 5,936 significant DE genes in bMDMs challenged with ST103 and 6443 DE genes in bMDMs challenged with ST12, compared to the respective unchallenged negative controls (FDR < 0.05 and *p* < 0.05). Moreover, 588 significant DE genes were detected when bMDMs challenged with ST12 were compared to bMDMs challenged with ST103 (FDR < 0.05 and *p* < 0.05; **Supplementary Table 4**).

The analyses of differential expression between low and high GEBV animals revealed one significant DE gene in bMDMs challenged with ST103, one significant DE gene in bMDMs challenged with ST12, and no significant genes in unchallenged negative controls. Furthermore, the analyses of differential expression between low and high SCC animals revealed six significant DE genes in bMDMs challenged with ST103, three significant DE genes in bMDMs challenged with ST12, and two significant DE genes in unchallenged controls (**Supplementary Table 5**).

### Enriched Pathways of Differentially Expressed Genes

**Figure 1:**
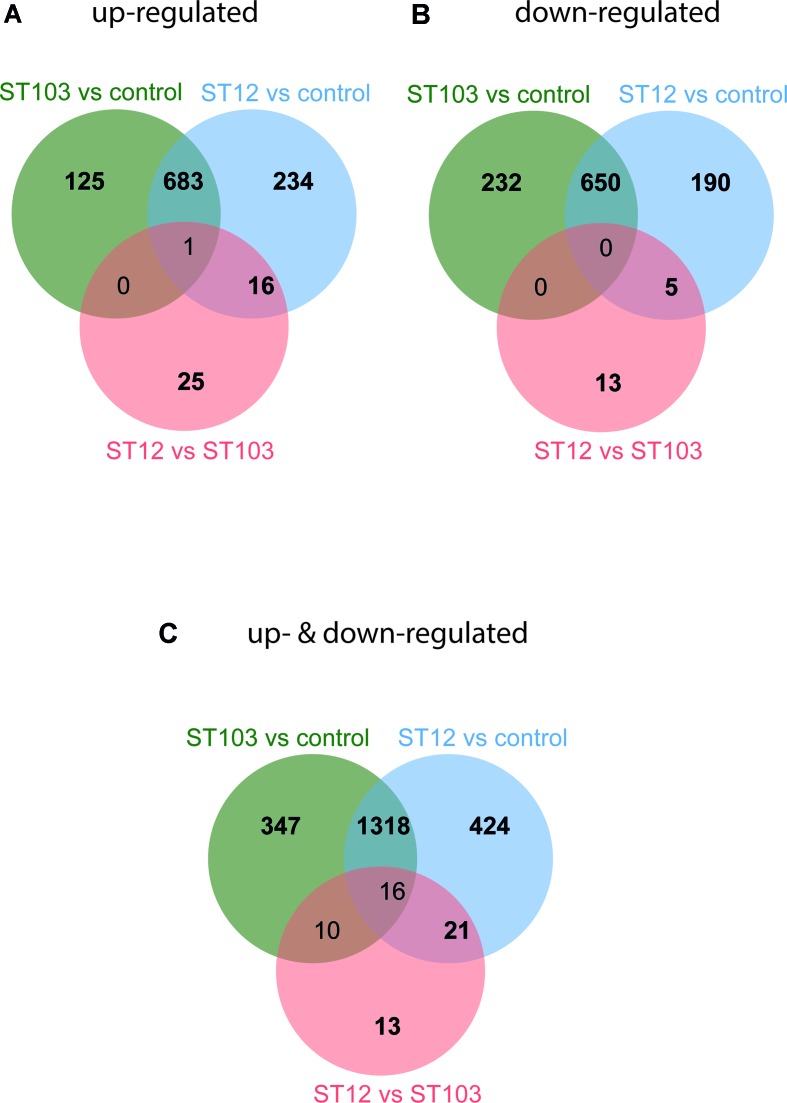
Venn diagrams of differentially expressed (DE) genes in bovine monocyte-derived macrophages (bMDMs) challenged with *Streptococcus agalactiae* strain ST103 or strain ST12, compared to the respective unchallenged negative controls, and in bMDMs challenged with strain ST12 compared to strain ST103 (FDR < 0.05). **(A)** Genes that were only up-regulated: log fold change (logFC) ≥ 1.5. **(B)** Genes that were only down-regulated: logFC ≤ −1.5. **(C)** Genes that were both up- and down-regulated: logFC ≥ 1.5 and logFC ≤ −1.5.

In order to identify the most biologically relevant DE genes, those with logFC either ≤−1.5 for down-regulated or ≥1.5 for up-regulated genes were identified and used in IPA (**Supplementary Table 4**). Venn diagrams for up-regulated and down-regulated genes identified above were constructed (**Figure 1**; **Supplementary Table 6**). The top significantly enriched canonical pathways identified by IPA are presented in **Table 1**.

**Table 1:** Top significant pathways overrepresented among differentially expressed (DE) genes in response to *in vitro* exposure of bovine monocyte-derived macrophages (bMDM) to *Streptococcus agalactiae* strain ST103 or strain ST12, as compared to negative controls.

		Pathway name	*P*-value	Genes
ST103 vs control	Up-regulated (809)	Differential Regulation of Cytokine Production in Macrophages and T Helper Cells by IL-17A and IL-17F	3,44E−11	IL9, CSF2, CCL5, TNF, CCL2, IL17A, IL10, IL12B, CSF3, CCL4
		Granulocyte Adhesion and Diapedesis	5,46E−10	GNAI1, CCL8, CLDN4, CCL20, HRH1, CCL3L3, SDC1, IL1RN, TNF, ITGB3, CCL4, SDC3, IL33, CCL5, CXCL10, EZR, CXCL2, HSPB1, CCL22, CCL2, CSF3, SDC4, ICAM1, IL36G, PECAM1
		Differential Regulation of Cytokine Production in Intestinal Epithelial Cells by IL-17A and IL-17F	7,78E−10	IL9, CSF2, CCL5, TNF, CCL2, IL17A, IL10, IL12B, CSF3, CCL4
	Down-regulated (882)	IL-8 Signaling	9,28E−08	PLCB2, GNG7, PLD1, ARRB2, IRAK3, VCAM1, NCF2, FOS, PIK3CD, RND3, VEGFC, PRKCE, LIMK2, ITGB5, ANGPT2, FLT4, PRKD3, PLD4, MYL9, PIK3CG, PIK3R2, CCND1, CXCR2, PLD2, CYBB
		Role of Pattern Recognition Receptors in Recognition of Bacteria and Viruses	3,49E−06	PRKCE, OAS1, IL12A, TLR7, PRKD3, NOD1, OAS2, C3AR1, C1QA, PIK3CG, C1QC, IL18, PIK3CD, TLR3, PIK3R2, C5AR1, CLEC6A, CLEC7A
		Phagosome Formation	2,76E−05	PRKCE, TLR7, PRKD3, PLCB2, MRC2, MSR1, C3AR1, PIK3CG, INPP5D, PIK3CD, TLR3, PIK3R2, C5AR1, ITGA4, RND3, CLEC7A
ST12 vs control	Up-regulated (934)	Th1 and Th2 Activation Pathway	9,08E−12	PRKCQ, JAK3, IL12RB2, GAB1, CD274, IL27, DLL4, IL9, STAT1, CD3D, STAT5A, IL12B, CRLF2, NFIL3, IL33, IL27RA, S1PR1, CD3E, STAT4, PIK3R3, JAG1, LTA, IL2RA, HLA-DOB, NFATC1, TNFSF4, DLL1, IL10, ICAM1, TBX21
		Differential Regulation of Cytokine Production in Macrophages and T Helper Cells by IL-17A and IL-17F	1,52E−10	CSF2, IL9, CCL5, TNF, CCL2, IL17A, IL12B, IL10, CSF3, CCL4
		Role of Hypercytokinemia/Hyperchemokinemia in the Pathogenesis of Influenza	2,88E−09	CCL5, CXCL10, IL37, CXCL8, IL9, IL1RN, TNF, CCL2, IL17A, IL12B, CCL4, IL36G, IL33
	Down-regulated (845)	IL-8 Signaling	5,55E−05	ITGB5, LIMK2, ANGPT2, FLT4, PLCB2, GNG7, MYL9, PLD4, PLD1, ARRB2, VCAM1, NCF2, FOS, PIK3CD, PIK3R2, CCND1, CXCR2, PLD2, RND3
		Role of Pattern Recognition Receptors in Recognition of Bacteria and Viruses	7,97E−05	OAS1, TLR7, C1QB, NOD1, OAS2, C3AR1, C1QA, C1QC, IL18, PIK3CD, TLR3, C5AR1, PIK3R2, CLEC6A, CLEC7A
		Complement System	2,36E−04	C1QC, C5AR1, C1QB, C7, CFD, C3AR1, C1QA
ST12 vs ST103	Up-regulated (42)	IL-12 Signaling and Production in Macrophages	1.30E−04	FOS, REL, PIK3R3, ALOX15
		Prolactin Signaling	4.36E−04	FOS, PIK3R3, TCF7
		Rac Signaling	1.18E−03	PIK3R3, CYFIP2, ABI2
	Down-regulated (18)	Thiosulfate Disproportionation III (Rhodanese)	1.78E−03	TST
		Th2 Pathway	3.46E−03	TNFSF4, IL2RA
		Granulocyte Adhesion and Diapedesis	4.89E−03	CCL8, CLDN4

The number in parentheses represents the number of DE genes used for Ingenuity Pathway Analyses (IPA).

**Figure 2:**
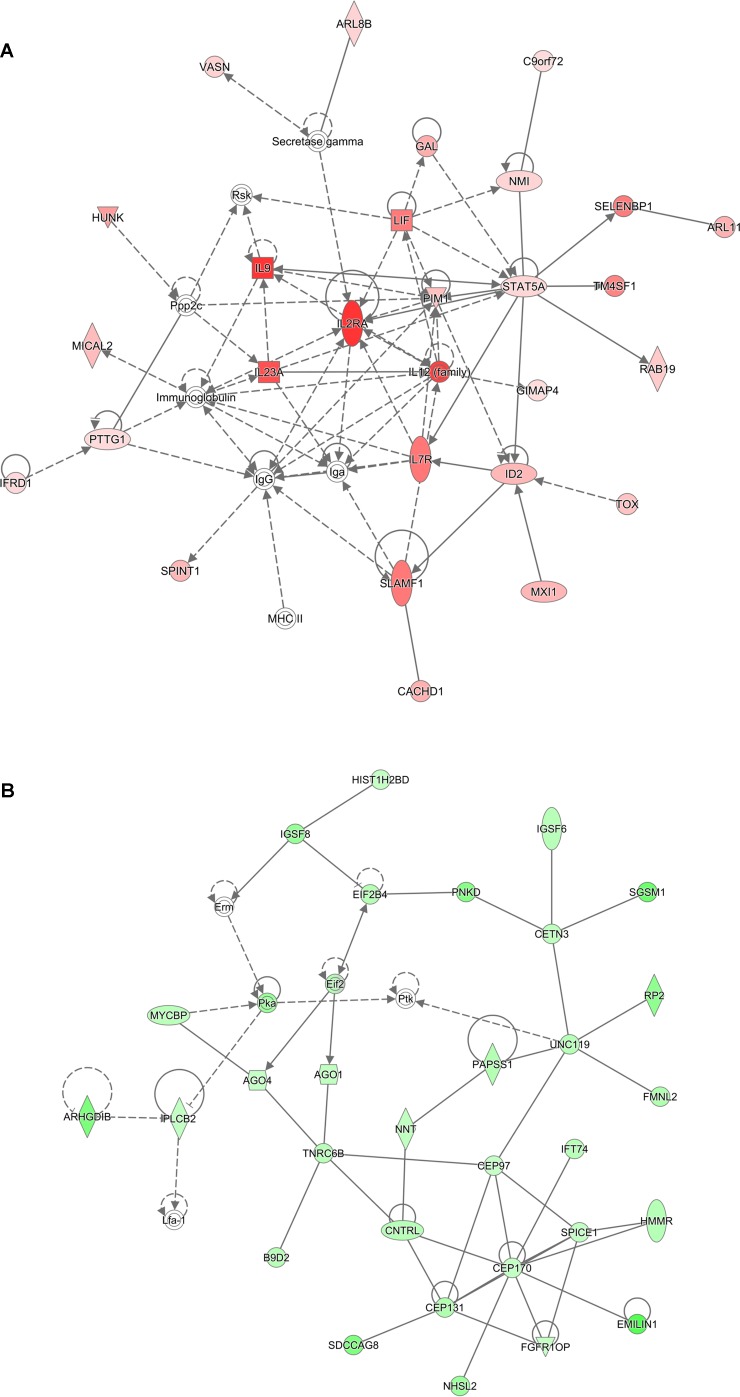
Functional networks overrepresented in the list of DE mRNA in response to infection of bMDMs with *Streptococcus agalactiae* strain ST103 compared to uninfected controls. Ingenuity Pathway Analysis (IPA) identified 27 associated molecules with a network score of 37 for **(A)** up-regulated genes and 30 associated molecules with a network score of 41 for **(B)** down-regulated genes.

**Figure 3:**
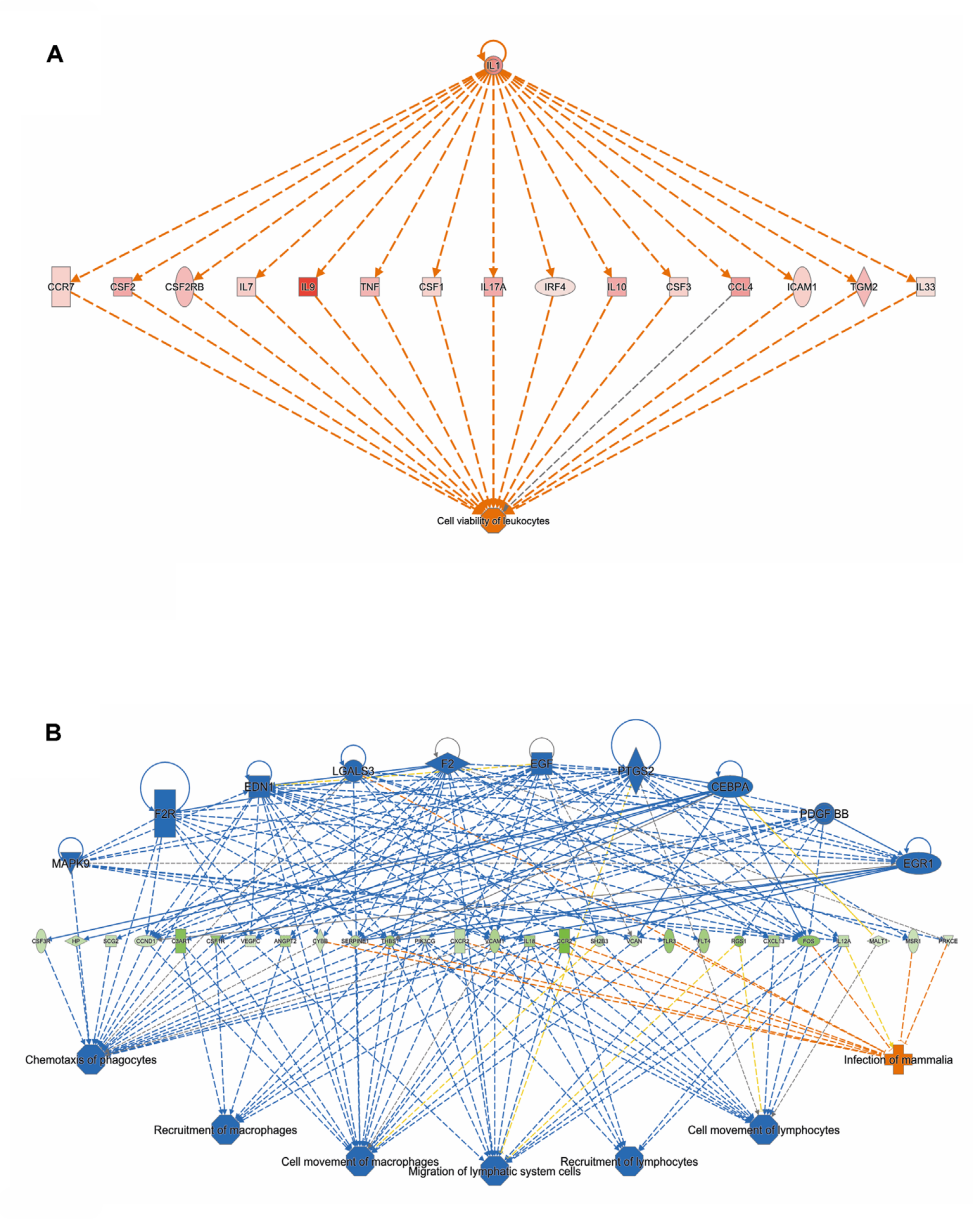
The top regulators identified by IPA **(A)** for genes up-regulated and **(B)** down-regulated in response to infection of bMDMs with *Streptococcus agalactiae* strain ST103 compared to uninfected controls.

Analysis of genes that were up-regulated in response to ST103 infection (*n* = 809) revealed that one of the top biological networks with 27 focus molecules was associated with cellular development, cellular growth and proliferation, hematological system development, and function (**Figure 2A**). Furthermore, *Interleukin 1* (*IL-1*) was identified as one of the top regulators affecting the viability of leukocytes (**Figure 3A**).

Analysis of ST103 down-regulated genes (*n* = 882) showed that one of the top biological networks with 30 focus molecules was associated with cell morphology, cellular assembly and organization, cellular function, and maintenance (**Figure 2B**). Moreover, top regulators affecting movement of leukocytes were identified among ST103 down-regulated genes (**Figure 3B**).

**Figure 4:**
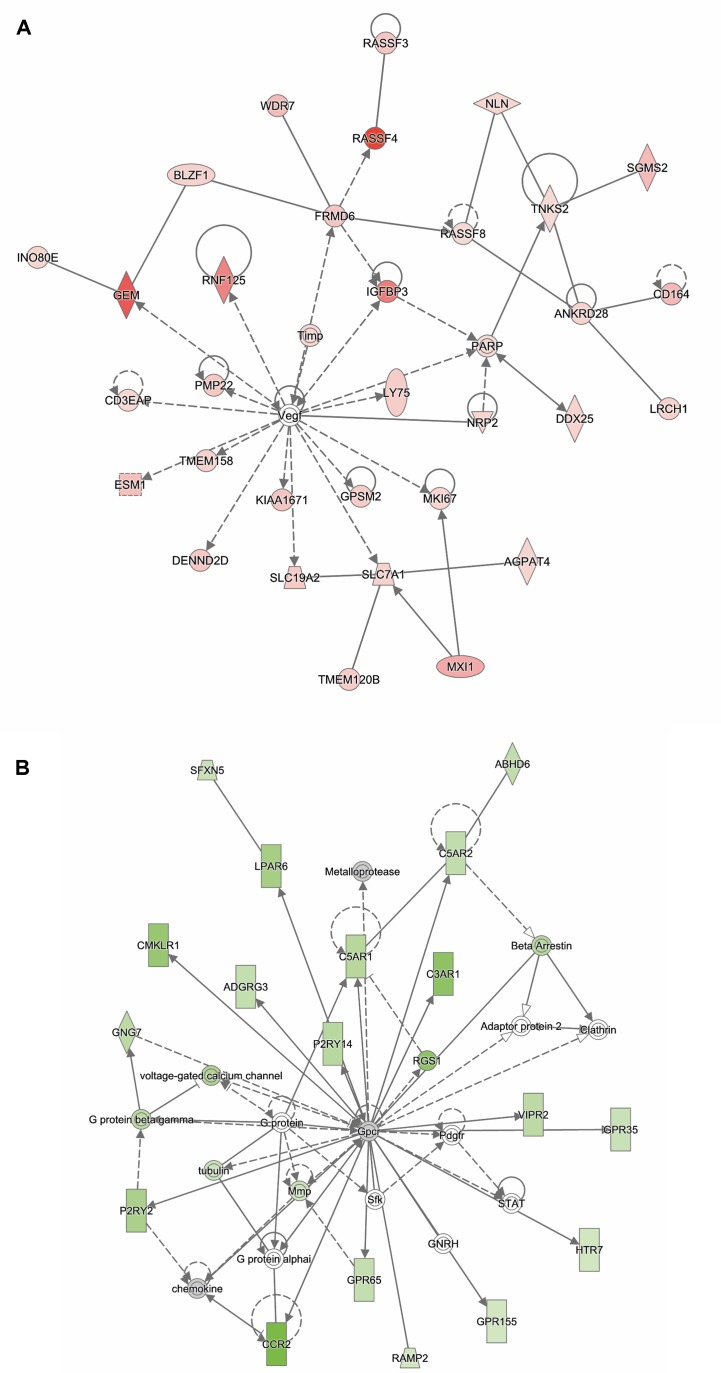
Functional networks overrepresented in the list of DE mRNA in response to infection of bMDMs with *Streptococcus agalactiae* strain ST12 compared to uninfected controls. IPA identified 32 associated molecules with a network score of 46 for **(A)** up-regulated genes and 19 associated molecules with a network score of 21 for **(B)** down-regulated genes.

Analysis of genes that were up-regulated in response to ST12 infection (*n* = 934) revealed that the top biological networks with 32 focus molecules was associated with cancer, organismal injury and abnormalities, and lipid metabolism (**Figure 4A**). Furthermore, *angiotensinogen* (*AGT*) was identified as one of the top regulators affecting recruitment of leukocytes (**Supplementary Figure 2**).

Analysis of ST12 down-regulated genes (*n* = 845) showed that one of the top biological networks with 19 focus molecules was associated with cell-to-cell signaling and interaction, cellular movement, hematological system development, and function (**Figure 4B**). Moreover, top regulators affecting movement of antigen-presenting cells were identified for ST12 down-regulated genes (**Supplementary Figure 3**).

**Figure 5:**
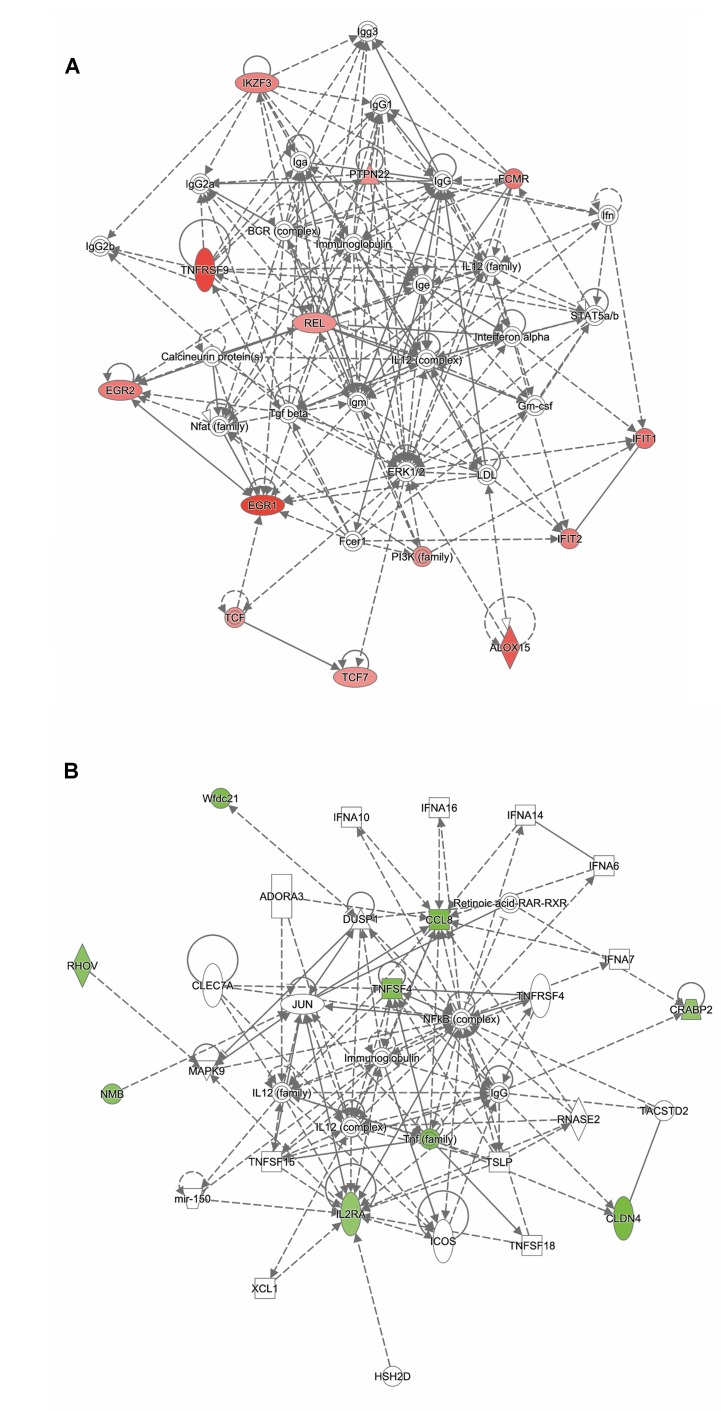
Functional networks overrepresented in the list of DE mRNA in response to infection of bMDMs with *Streptococcus agalactiae* strain ST12 compared to infection with strain ST103. IPA identified 11 associated molecules with a network score of 23 for **(A)** up-regulated genes and eight associated molecules with a network score of 20 for **(B)** down-regulated genes.

Analysis of genes up-regulated in ST12 compared to ST103 (*n* = 42) revealed that the top biological network with 13 focus molecules was associated with cell-mediated immune response, cellular development, cellular function, and maintenance (**Figure 5A**). Moreover, the top regulators involved in apoptosis of lymphocytes and inflammatory response were identified for these up-regulated genes (**Supplementary Figure 4**).

Analysis of genes down-regulated in ST12 compared to ST103 (*n* = 18) revealed that the top biological network with eight focus molecules was associated with hematological system development and function, immune cell trafficking, and inflammatory response (**Figure 5B**). There were no top regulators identified for these genes.

All detailed information from IPA are presented in **Supplementary Table 7**.

## Discussion

Transcription profiles of macrophages infected with several types of bacteria and bacterial and viral ligands have been studied earlier (Lewandowska-Sabat et al., 2013; Nalpas et al., 2013; Casey et al., 2015; Mavromatis et al., 2015; Hop et al., 2017; Roy et al., 2018; Toca et al., 2019). To the best of our knowledge, this is the first study of the early-phase transcriptome of bMDMs infected *in vitro* with two strains of *S. agalactiae*. *S. agalactiae* is not a main causative agent of subclinical mastitis in Norway, but its occurrence has increased in modern freestalls and automatic milking systems the last years. The agent is also a main cause of life-threatening infections in human neonates, pregnant females, and elderly adults (Henneke and Berner, 2006). The two strains used in this study vary in their capability to survive in the environment and transmit within dairy herds. ST103 is the most prevalent and persistent isolate found in bovine herds with substantial environmental contamination, while ST12 was found in cattle herds with no positive environmental samples (Jorgensen et al., 2016). We have analyzed over 1,648 million reads and found several thousand transcripts that were significantly differentially regulated in response to infection (**Supplementary Table 4**). In order to identify the most biologically relevant genes, the logFC threshold was set to ≥1.5 for up-regulated and ≤−1.5 for down-regulated transcripts (**Figure 1**), and pathways analyses of these transcripts were performed.

We observed that ST103 induced a different transcript profile during the early stage of macrophage infection compared to ST12 (**Figure 1** and **Table 1**). The observed differences may be explained by differences in virulence factors and/or the different niches and modes of transmission of these strains. It has been demonstrated that specific sequence types of bacteria often associate with specific virulence factors, which, in turn, are associated with the outcome of infection in the host (Tassi et al., 2015; Budd et al., 2016). The virulence factors in strain ST103 are associated with growth ability in milk, biofilm formation, and adhesion to bovine mammary epithelial cells as well as higher cytotoxicity compared to most of the other bovine strains (Pang et al., 2017). On the other hand, the characterization of ST12 revealed that this strain can survive within macrophages (Korir et al., 2017).

Analyses of global transcriptomics and gene networks have been performed earlier in livestock in order to reveal candidate genes and pathways (Kogelman et al., 2014; Drag et al., 2017). One of the top canonical pathways for the up-regulated transcripts in ST103-infected macrophages was granulocyte adhesion and diapedesis (**Table 1**). During host defense against infection, the polymorphonuclear neutrophils and macrophages are rapidly recruited to sites of bacterial invasion. This multi-step process involves an action of several chemokines and their ligands mediating the initiation of contact between granulocytes and endothelial cells. Integrins are cell adhesion molecules involved in this process, and many bacterial species, such as group A streptococci (Ozeri et al., 2001), *S. aureus* (Agerer et al., 2005), and *E. coli* (Plancon et al., 2003), use host integrins for adhering to and invading host cells. Furthermore, a recent study has identified virulence factors in strain ST103 of *S. agalactiae* associated with ability to adhere to bovine mammary epithelial cells (Pang et al., 2017). Interestingly, a recent study from our group showed that target genes of microRNAs that were down-regulated in ST103-infected bovine macrophages were associated with the integrin signaling pathway (Lewandowska-Sabat et al., 2018a). These findings, together with the present results, may indicate that granulocyte adhesion is crucial for invasion of macrophages or mammary epithelial cells by *S. agalactiae* strain ST103. Furthermore, one of the most central molecules in the network associated with the response to strain ST103 was *interleukin-9* (*IL-9*; **Figure 2A**). This cytokine stimulates cell proliferation and prevents apoptosis, is associated with type 2 immune responses, and has been identified as a susceptibility factor in bacterial infection (Arendse et al., 2005; Wynn, 2015). In recent years, type 2 immune response has emerged as a major effector response either acting in a host protective manner or having pathogenic activity (Wynn, 2015). It has been shown that type 2 cytokine responses suppress the development of protective type 1 immunity to bacterial pathogens and facilitate uncontrolled or persistent infection (Erb et al., 1998; Harris et al., 2007). Although type 2 immune response enables tissue repair, prolonged activation of this pathway may contribute to the development of pathological conditions as it is observed in many chronic fibroproliferative diseases (Wynn and Ramalingam, 2012). We have also identified several molecules associated with cell viability of leukocytes (**Figure 3A**). It has been demonstrated recently that *S. agalactiae* survive intracellularly inducing injury to murine macrophages (Guo et al., 2014) and that an increased expression of anti-apoptotic genes in *Staphylococcus aureus* infected human macrophages contributes to extended phagocyte lifetime allowing intracellular bacterial survival (Koziel et al., 2009). These observations, together with our findings, may suggest that decreased apoptosis and amplified type 2 immune response may likely be an evasion strategy developed by *S. agalactiae* strain ST103 to avoid host defense and promote its intracellular replication and persistence.

One of the top canonical pathways for the down-regulated transcripts in ST103-infected macrophages was phagosome formation (**Table 1**). In addition, cellular assembly and organization was identified as one of the most significant networks associated with the down-regulated transcripts (**Figure 2B**). Phagocytosis is driven by a tightly controlled rearrangement of the actin cytoskeleton to form the phagosome upon recognition of pathogens, and it has been shown that some bacteria, e.g. *Mycobacterium tuberculosis* (Wong et al., 2013), *Listeria monocytogenes* (Portnoy et al., 2002), and *Group B Streptococcus* (Korir et al., 2017), can exploit phagocytosis as an evasion strategy. Furthermore, interleukin-8 (IL-8) signaling pathway (**Table 1**) and movement of leukocytes (**Figure 3B**) were recognized as the most significant pathways and regulator effects, respectively, for the ST103 down-regulated transcripts in bMDMs. It has been shown that monocyte recruitment is critical for immune defense against a broad range of pathogens (Peters et al., 2001; Pamer, 2004; Copin et al., 2007). These results suggest that inhibition of chemotaxis of other immune cells, e.g., neutrophils to the site of infection, may be used by strain ST103 in order to either evade or suppress host immune response. These hypotheses need to be confirmed, however, by functional studies.

The most significant canonical pathway identified among the up-regulated transcripts in ST12-infected macrophages was the type 1/type 2 T helper cell (Th1/Th2) activation pathway (**Table 1**). In addition, many genes associated with the lipid metabolism network were identified (**Figure 4A**). While M1 macrophages produce *IL-12* and constitute the first line of defense against pathogens, M2 macrophages appear as a favorable environment for long-term persistence of many pathogens (reviewed in Muraille et al., 2014). Several studies suggest that some intracellular bacteria such as *Brucella abortus* and *Mycobacterium tuberculosis* responsible for chronic infections actively manipulate signal transducer and activator of transcription 6 (STAT6)–peroxisome proliferator activated receptor gamma/delta (PPARγ/δ) pathways to circumvent M1 macrophage polarization and benefit from a nutrient-rich niche associated with lipid metabolism (Jensen et al., 2011; Xavier et al., 2013; Almeida et al., 2014). In our previous study, we demonstrated that both bacterial strains regulate miRNAs involved in the alternative activation of macrophages (Lewandowska-Sabat et al., 2018a). This may suggest that both strains of *S. agalactiae* may induce alternative macrophage activation; however, ST12 seems to contribute to M2 polarization to a larger extent than ST103. This result, however, needs to be confirmed by more comprehensive functional studies.

Complement system activation was one of the top canonical pathways identified among the down-regulated transcripts in the ST12-infected macrophages (**Table 1**). The complement system is a central component of innate immunity, produced by several cellular sources, including macrophages (Lubbers et al., 2017). Several microorganisms have developed many ways to evade complement actions (reviewed in Lambris et al., 2008). Several of the genes of the complement system were down-regulated in bMDMs in response to ST12 infection (**Table 1** and **Figure 4B**), which indicate that this may be one of the mechanisms developed by ST12 to escape the attack of complement.

Analysis of differential gene response between ST103- and ST12-infected bMDMs revealed that IL-12 signaling and production in macrophages (**Table 1**) and gene network associated with cell-mediated immune response (**Figure 5A**) were significantly up-regulated in ST12 compared to ST103. *Fos proto-oncogene* (*FOS*), *early growth response 1* (*EGR1*), and *early growth response 2* (*EGR2*) are transcription factors known as immediate-early genes (IEGs), i.e., they are able to respond very quickly to regulatory signals, for example, in immune responses (reviewed in Bahrami and Drablos, 2016). It has been demonstrated that stimulation of mouse bone marrow-derived macrophages with lipopolisacharide leads to strong induction of these genes (Ramirez-Carrozzi et al., 2009). *FOS* plays a key role in proliferation, differentiation, and survival of the cell (O’Donnell et al., 2012), and *EGR1* promotes expression of *IL-8*, *interleukin-6* (*IL-6*), and *tumor necrosis factor* (*TNF*) by binding to their promoter region (de Klerk et al., 2017). *FOS*, *EGR1*, and *EGR2* are significantly up-regulated in ST12- compared to ST103-infected bMDMs. These results may suggest that infection of bMDMs by ST12 promotes gene responses crucial for bacterial intracellular survival. Moreover, suppression of these transcription factors in ST103-infected cells may possibly constitute a strategy to evade an early immune response and favor persistence of this *S. agalactiae* strain.

One of the top canonical pathways identified for down-regulated genes in ST12- compared to ST103-infected bMDMs was granulocyte adhesion and diapedesis (**Table 1**). This pathway was also significantly overrepresented among up-regulated genes in ST103-infected bMDMs compared to uninfected controls, which suggests that ST103 modifies expression of genes involved in cell adhesion in order to invade the macrophages.

*Myosin IF* (*MYO1F*) was found to be significantly up-regulated in ST103-infected bMDMs from animals with low compared to high GEBV for SCC (**Supplementary Table 5**). It has been demonstrated recently that *MYO1F* plays an important role in neutrophil trafficking during inflammation by regulating neutrophil extravasation and migration (Salvermoser et al., 2018). This suggests that low GEBV animals, i.e., those with a higher probability of having high SCC in milk, have increased *MYO1F* expression that facilitates neutrophil migration to the infected udder during mastitis, which, in turn, may explain high SCC in milk. *MYO1F* is a possible functional candidate gene for susceptibility to subclinical mastitis and high SCC in Norwegian Red; however, functional studies are required to verify this hypothesis.

*Peptidyl arginine deiminase 4* (*PADI4*) was found to be significantly down-regulated in ST12-infected bMDMs from animals with low compared to high GEBV for SCC (**Supplementary Table 5**). It has been shown that *PADI4* mediates chromatin decondensation in neutrophils in response to inflammatory stimuli (Neeli et al., 2008). This suggests the possibility that *PADI4* might display a role in the susceptibility to subclinical mastitis and that low GEBV animals may have impaired neutrophil responses to infection. However, this hypothesis needs to be further tested.

A few genes were found to be differentially expressed between high and low SCC animals (**Supplementary Table 5**). *Defensin beta 5* (*DEFB5*) was down-regulated in both ST103- and ST12-infected bMDMs, while *C-X-C motif chemokine receptor 2* (*CXCR2*) was down-regulated only in ST103-infected bMDMs from animals with high compared to low SCC. Bovine beta-defensins are antimicrobial peptides providing first-line protection against pathogens, and their importance in host defense against mastitis-causing pathogens has been demonstrated (reviewed in Meade et al., 2014). The lower abundance of *DEFB5* mRNA observed in our study in bMDMs from high compared to low SCC animals may be associated with delayed clearance of bacteria from the udder during infection. *CXCR2* regulates neutrophil recruitment in a number of pathological conditions (Del Rio et al., 2001). It has been recently demonstrated that CXCR2-deficient mice display pro-inflammatory responses and increase in macrophage accumulation at inflamed sites during acute inflammation (Dyer et al., 2017). The observed lower abundance of *CXCR2* mRNA in bMDMs from high compared to low SCC animals can indicate a higher level of inflammatory response in these animals, resulting in a high number of SCC in milk. Additionally, *macrophage receptor with collagenous structure* (*MARCO*) was up-regulated in ST103-infected, ST12-infected, and uninfected control bMDMs from animals with high compared to low SCC. *MARCO* is a scavenger receptor that enables recruitment of mononuclear cells and pro-inflammatory cytokine production in response to bacterial infection (Areschoug and Gordon, 2009; Dorrington et al., 2013). Interestingly, this gene was found 4.8 Mb from a single nucleotide polymorphism (SNP) significantly associated with high SCC in Norwegian Red (Kirsanova et al. in prep). Higher abundance of *MARCO* mRNA in bMDMs from high compared to low SCC animals may be associated with higher level of pro-inflammatory response and macrophage accumulation in high SCC animals. *MARCO* mRNA level was higher in unstimulated control bMDMs from high compared to low SCC animals. This indicates that transcription of this gene in monocytes is triggered by a pathogen infection and remains at the high level even long after the exposure. This may point toward *MARCO* as a potential biomarker for persistent chronic mastitis.

Whereas high milk SCC is a mastitis-related phenotype, the animals with low GEBV for SCC are likely to develop a high milk SCC phenotype, though it is not always the case. This indicates that the differentially expressed genes between low and high GEBV animals may be putative candidates for susceptibility to subclinical mastitis in Norwegian Red. However, comprehensive functional studies, both *in vitro* and *in vivo*, are necessary in order to verify all of these findings before including information on genetic variants of these genes in the genomic selection schemes. It is worth noting that even though the methods used for estimation of the differential expression (edgeR and DESeq2) perform well and are recommended for these type of analyses (Schurch et al., 2016), it may still be likely that some of these genes are false positives. Furthermore, it is important to mention that the results presented in our study are based on relative low sample size, which can, in turn, affect the outcomes of the analyses.

In conclusion, in our study, several genes and pathways were identified as differentially expressed during an early stage of bMDM infection with two *S. agalactiae* strains. Genes of granulocyte adhesion and phagosome formation pathways were significantly regulated in ST103-infected bMDMs, which may indicate the essential strategy of this strain to avoid host defense and promote its intracellular replication and persistence. Genes of Th1/Th2 activation and complement activation pathways were affected by ST12 infection, suggesting putative mechanisms developed by ST12 to escape the attack of the immune system. Furthermore, significant differences in gene responses between ST103 and ST12 have been identified, such as IL-12 signaling and granulocyte adhesion, which may partly be explained by differences in virulence factors between these strains. However, whether these pathways are evasion strategies developed by *S. agalactiae* to avoid host defense and promote its intracellular survival remains to be determined by functional studies. We have also identified *MARCO* as a potential biomarker for persistent chronic mastitis, and *MYO1F* and *PADI4* as putative candidates for susceptibility to subclinical mastitis in Norwegian Red. These results contribute to understanding of mechanisms involved in the pathogenesis of subclinical mastitis.

## Ethics Statement

Blood sampling was performed by certified personnel and conducted in accordance with the laws and regulations controlling experiments using live animals in Norway. The study was approved by the Norwegian Animal Research Authority (Norwegian Food Safety Authority; FOTS id: 8661).

## Author Contributions

AL-S participated in the design of the study, carried out the infection experiments, analyzed the RNA sequencing data, performed the pathway analyses, and drafted the manuscript. EK analyzed the RNA sequencing data and participated in the part of the infection experiment and the manuscript drafting. CK provided software and participated in the RNA sequencing data analyses and manuscript drafting. PB, TS, BH, OØ, and IO participated in the study design, discussion, and interpretation of the results and manuscript drafting. All authors read and approved the final version of the manuscript.

## Funding

This paper is a part of the “Multimast” project (no. 233778) funded by the Research Council of Norway, Tine and Geno.

## Conflict of Interest Statement

Authors TS and BH were employed by company Geno SA. The remaining authors declare that the research was conducted in the absence of any commercial or financial relationships that could be construed as a potential conflict of interest.
