# Supplementary material for: Transcription Profiling of Monocyte-Derived Macrophages Infected In Vitro With Two Strains of Streptococcus agalactiae Reveals Candidate Pathways Affecting Subclinical Mastitis in Cattle
Source: Front Genet. 2019 Jul 26;10:689. doi: 10.3389/fgene.2019.00689 (PMC6681682; doi:10.3389/fgene.2019.00689)
Supplement: Supplementary file 1 [file Table_1.docx]

| Animal ID | Farm ID | GEBV on SCC | SCC (cells/ml) | Grouping GEBV | Grouping SCC | Farm type |
| --- | --- | --- | --- | --- | --- | --- |
| 1 | 1 | 93 | 754,000 | Low | High | AMS/F |
| 2 | 1 | 114 | 15,000 | High | Low | AMS/F |
| 3 | 2 | 95 | 15,000 | Low | Low | AMS/F |
| 4 | 2 | 97 | 18,000 | Low | Low | AMS/F |
| 5 | 2 | 105 | 18,000 | High | Low | AMS/F |
| 6 | 2 | 109 | 18,000 | High | Low | AMS/F |
| 7 | 1 | 90 | 779,000 | Low | High | AMS/F |
| 8 | 1 | 93 | 667,000 | Low | High | AMS/F |
| 9 | 1 | 107 | 18,000 | High | Low | AMS/F |
| 10 | 3 | 97 | 13,000 | Low | Low | CMS/F |
| 11 | 3 | 120 | 16,000 | High | Low | CMS/F |
| 12 | 3 | 122 | 16,000 | High | Low | CMS/F |

**Supplementary Table 1.**

The animals used in the study.

GEBV – genomic estimated breeding value, SCC – somatic cell count, SCC (cells/ml) – geometric mean of three measurements of somatic cell count in milk during the last lactation, AMS – automatic milking system, F – freestall, CMS – conventional milking system.
