# Supplementary material for: Transcription Profiling of Monocyte-Derived Macrophages Infected In Vitro With Two Strains of Streptococcus agalactiae Reveals Candidate Pathways Affecting Subclinical Mastitis in Cattle
Source: Front Genet. 2019 Jul 26;10:689. doi: 10.3389/fgene.2019.00689 (PMC6681682; doi:10.3389/fgene.2019.00689)
Supplement: Supplementary file 2 [file Table_2.docx]

**Supplementary Table 2.**

Summary of the results from Illumina sequencing of bovine monocyte-derived macrophages (bMDMs) transcriptome and alignment to the bovine reference genome Bos taurus v3.1. Control – negative control: uninfected bMDMs; ST103 and ST12 – bMDMs infected with the ST103 or the ST12 strain of *Streptococcus agalactiae*, respectively.

| Sample name | Total number of reads (paired end) | Total number of reads passing filters (percentage) | Percentage of uniquely mapped reads |
| --- | --- | --- | --- |
| 1-RNA1-control | 46,716,220 | 46,522,156 (99.6%) | 85.73% |
| 2-RNA1-ST12 | 55,211,689 | 54,841,861 (99.3%) | 86.77% |
| 3-RNA1-ST103 | 43,583,615 | 43,416,422 (99.6%) | 86.79% |
| 4-RNA2-control | 46,453,702 | 46,130,305 (99.3%) | 79.27% |
| 5-RNA2-ST12 | 57,132,859 | 56,924,239 (99.6%) | 86.89% |
| 6-RNA2-ST103 | 44,305,652 | 44,124,250 (99.6%) | 86.66% |
| 7-RNA4-control | 43,210,326 | 42,942,817 (99.4%) | 87.42% |
| 8-RNA4-ST12 | 49,028,231 | 48,819,816 (99.6%) | 86.67% |
| 9-RNA4-ST103 | 54,733,215 | 54,497,956 (99.6%) | 78.89% |
| 10-RNA5-control | 59,267,812 | 58,930,499 (99.4%) | 87.53% |
| 11-RNA5-ST12 | 41,598,991 | 41,246,491 (99.2%) | 89.13% |
| 12-RNA5-ST103 | 40,005,853 | 39,512,928 (98.8%) | 82.61% |
| 13-RNA6-control | 56,282,438 | 55,966,732 (99.4%) | 88.02% |
| 14-RNA6-ST12 | 31,101,943 | 30,768,233 (98.9%) | 85.72% |
| 15-RNA6-ST103 | 35,049,353 | 34,698,255 (99.0%) | 87.96% |
| 16-RNA7-control | 43,382,792 | 43,144,051 (99.4%) | 86.73% |
| 17-RNA7-ST12 | 69,204,326 | 68,743,984 (99.3%) | 87.70% |
| 18-RNA7-ST103 | 67,310,229 | 66,574,650 (98.9%) | 88.14% |
| 19-RNA8-control | 41,369,950 | 41,173,893 (99.5%) | 87.98% |
| 20-RNA8-ST12 | 37,210,508 | 37,090,345 (99.7%) | 84.42% |
| 21-RNA8-ST103 | 34,940,864 | 34,839,101 (99.7%) | 87.77% |
| 22-RNA9-control | 36,572,962 | 36,439,529 (99.6%) | 85.89% |
| 23-RNA9-ST12 | 44,110,490 | 43,971,700 (99.7%) | 89.15% |
| 24-RNA9-ST103 | 39,831,179 | 39,744,268 (99.8%) | 89.11% |
| 25-RNA10-control | 52,317,464 | 52,151,716 (99.7%) | 88.14% |
| 26-RNA10-ST12 | 66,448,527 | 66,008,925 (99.3%) | 86.96% |
| 27-RNA10-ST103 | 49,827,661 | 49,656,291 (99.7%) | 87.93% |
| 28-RNA11-control | 39,688,837 | 39,511,757 (99.6%) | 80.72% |
| 29-RNA11-ST12 | 46,690,018 | 46,539,724 (99.7%) | 83.38% |
| 30-RNA11-ST103 | 36,320,764 | 36,104,819 (99.4%) | 82.45% |
| 31-RNA12-control | 37,743,793 | 37,634,899 (99.7%) | 87.31% |
| 32-RNA12-ST12 | 44,230,782 | 44,119,535 (99.7%) | 87.09% |
| 33-RNA12-ST103 | 44,485,208 | 44,406,157 (99.8%) | 88.21% |
| 34-RNA13-control | 33,538,407 | 33,372,974 (99.5%) | 86.31% |
| 35-RNA13-ST12 | 52,134,761 | 51,948,766 (99.6%) | 88.43% |
| 36-RNA13-ST103 | 35,726,168 | 35,571,863 (99.6%) | 86.91% |
