# Supplementary material for: Transcription Profiling of Monocyte-Derived Macrophages Infected In Vitro With Two Strains of Streptococcus agalactiae Reveals Candidate Pathways Affecting Subclinical Mastitis in Cattle
Source: Front Genet. 2019 Jul 26;10:689. doi: 10.3389/fgene.2019.00689 (PMC6681682; doi:10.3389/fgene.2019.00689)
Supplement: Supplementary file 3 [file Table_3.docx]

**Supplementary Table 3.**

The RNA samples used in the study.

| Sample ID | Animal ID | Conc. (ng/µl) | A260/280 | RIN |
| --- | --- | --- | --- | --- |
| RNA1_control | 1 | 75,8 | 2,1 | 8,8 |
| RNA1_ST12 | 1 | 74,1 | 2,1 | 9,2 |
| RNA1_ST103 | 1 | 45,5 | 2,2 | 8,7 |
| RNA2_control | 2 | 85,5 | 2,1 | 9,0 |
| RNA2_ST12 | 2 | 60,4 | 1,9 | N/A |
| RNA2_ST103 | 2 | 32,8 | 2,0 | 9,0 |
| RNA4_control | 3 | 111,8 | 2,0 | 8,5 |
| RNA4_ST12 | 3 | 62,9 | 2,2 | 8,4 |
| RNA4_ST103 | 3 | 49,2 | 2,0 | 9,0 |
| RNA5_control | 4 | 35,5 | 2,2 | 9,2 |
| RNA5_ST12 | 4 | 38 | 2,0 | 8,3 |
| RNA5_ST103 | 4 | 25,5 | 2,8 | 8,7 |
| RNA6_control | 5 | 61,6 | 2,1 | 8,8 |
| RNA6_ST12 | 5 | 24,7 | 2,0 | 8,6 |
| RNA6_ST103 | 5 | 22,6 | 2,1 | 8,8 |
| RNA7_control | 6 | 28 | 2,1 | 9,0 |
| RNA7_ST12 | 6 | 35,5 | 1,8 | 8,2 |
| RNA7_ST103 | 6 | 23 | 1,7 | 9,1 |
| RNA8_control | 7 | 18,9 | 2,0 | 8,9 |
| RNA8_ST12 | 7 | 18,9 | 2,0 | 7,7 |
| RNA8_ST103 | 7 | 10,1 | 2,3 | N/A |
| RNA9_control | 8 | 63,5 | 2,2 | 8,6 |
| RNA9_ST12 | 8 | 40,5 | 2,2 | N/A |
| RNA9_ST103 | 8 | 57,7 | 2,2 | 8,7 |
| RNA10_control | 9 | 16,2 | 2,0 | N/A |
| RNA10_ST12 | 9 | 24,3 | 2,0 | N/A |
| RNA10_ST103 | 9 | 13,4 | 2,7 | N/A |
| RNA11_control | 10 | 80,4 | 2,0 | 9,4 |
| RNA11_ST12 | 10 | 35,6 | 2,2 | 4,0 |
| RNA11_ST103 | 10 | 104,2 | 2,1 | 7,2 |
| RNA12_control | 11 | 110,7 | 2,0 | 9,9 |
| RNA12_ST12 | 11 | 38,9 | 2,0 | N/A |
| RNA12_ST103 | 11 | 72,9 | 2,0 | N/A |
| RNA13_control | 12 | 79,1 | 2,1 | 9,5 |
| RNA13_ST12 | 12 | 34,8 | 2,3 | N/A |
| RNA13_ST103 | 12 | 66,7 | 2,1 | N/A |

RIN - RNA integrity number, N/A – not available.
