# Supplementary figures and images for: Transcription Profiling of Monocyte-Derived Macrophages Infected In Vitro With Two Strains of Streptococcus agalactiae Reveals Candidate Pathways Affecting Subclinical Mastitis in Cattle
Source: Front Genet. 2019 Jul 26;10:689. doi: 10.3389/fgene.2019.00689 (PMC6681682; doi:10.3389/fgene.2019.00689)

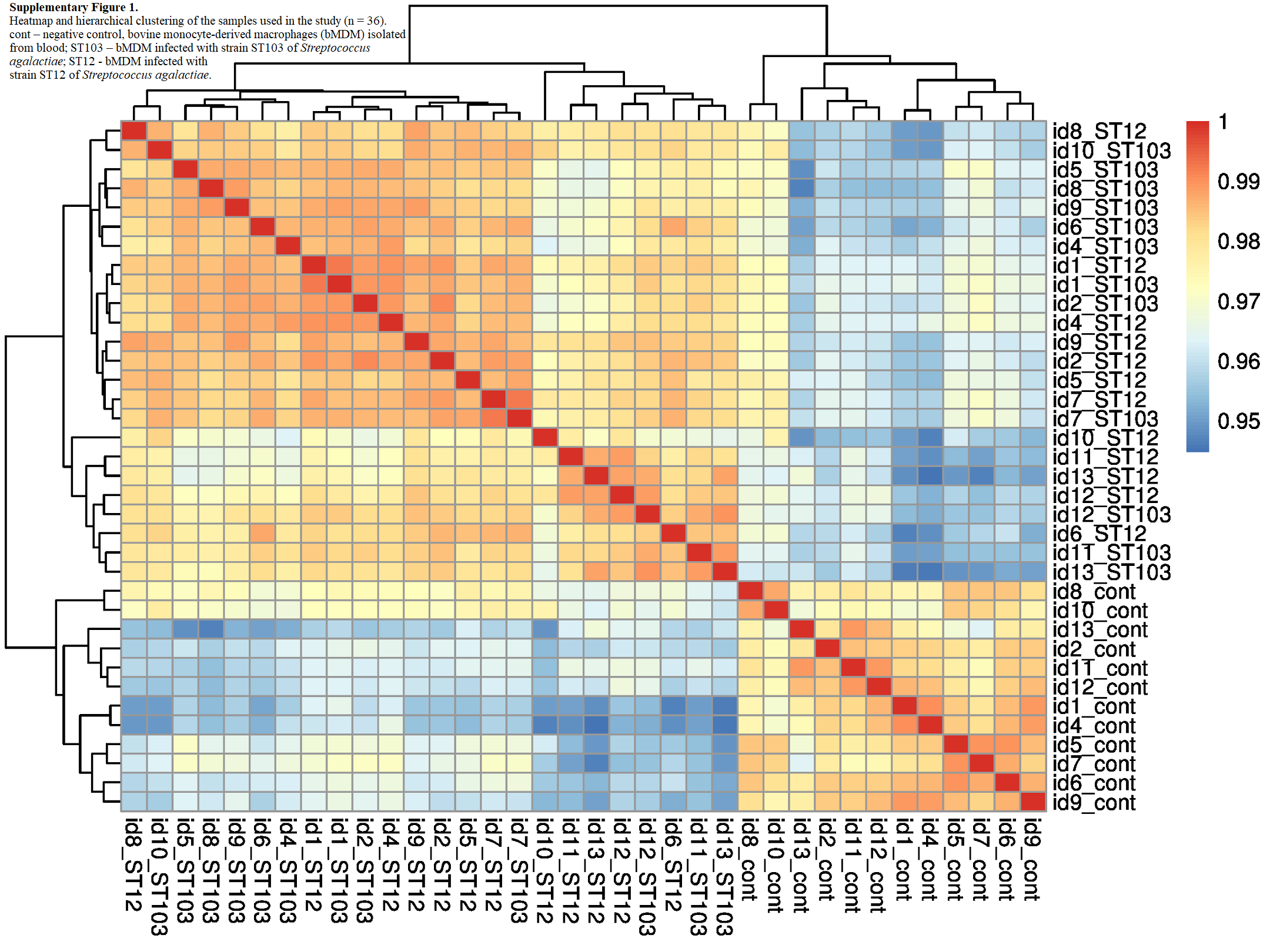

Supplement: Supplementary file 8 [file Image_1.tiff]

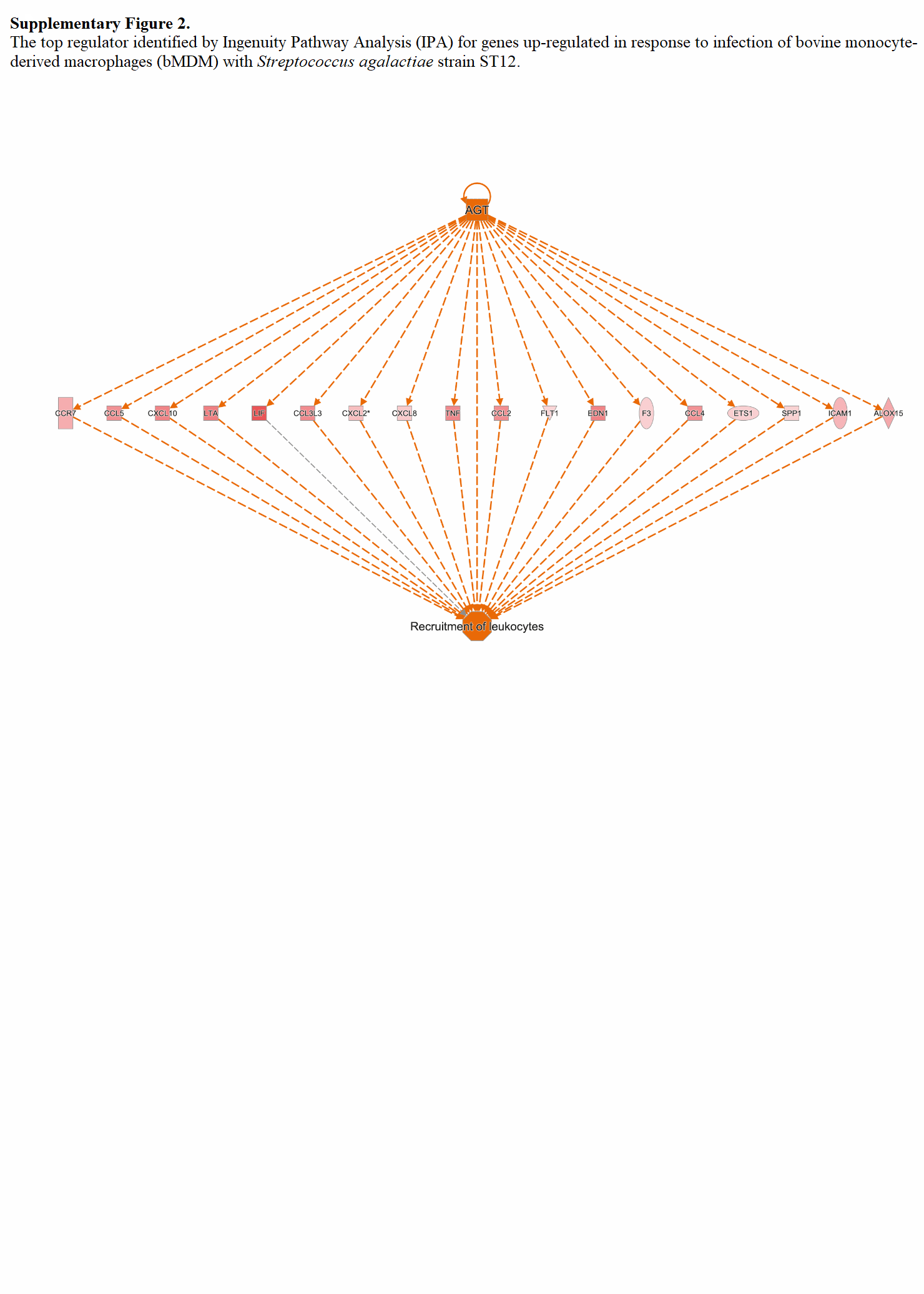

Supplement: Supplementary file 9 [file Image_2.tif]

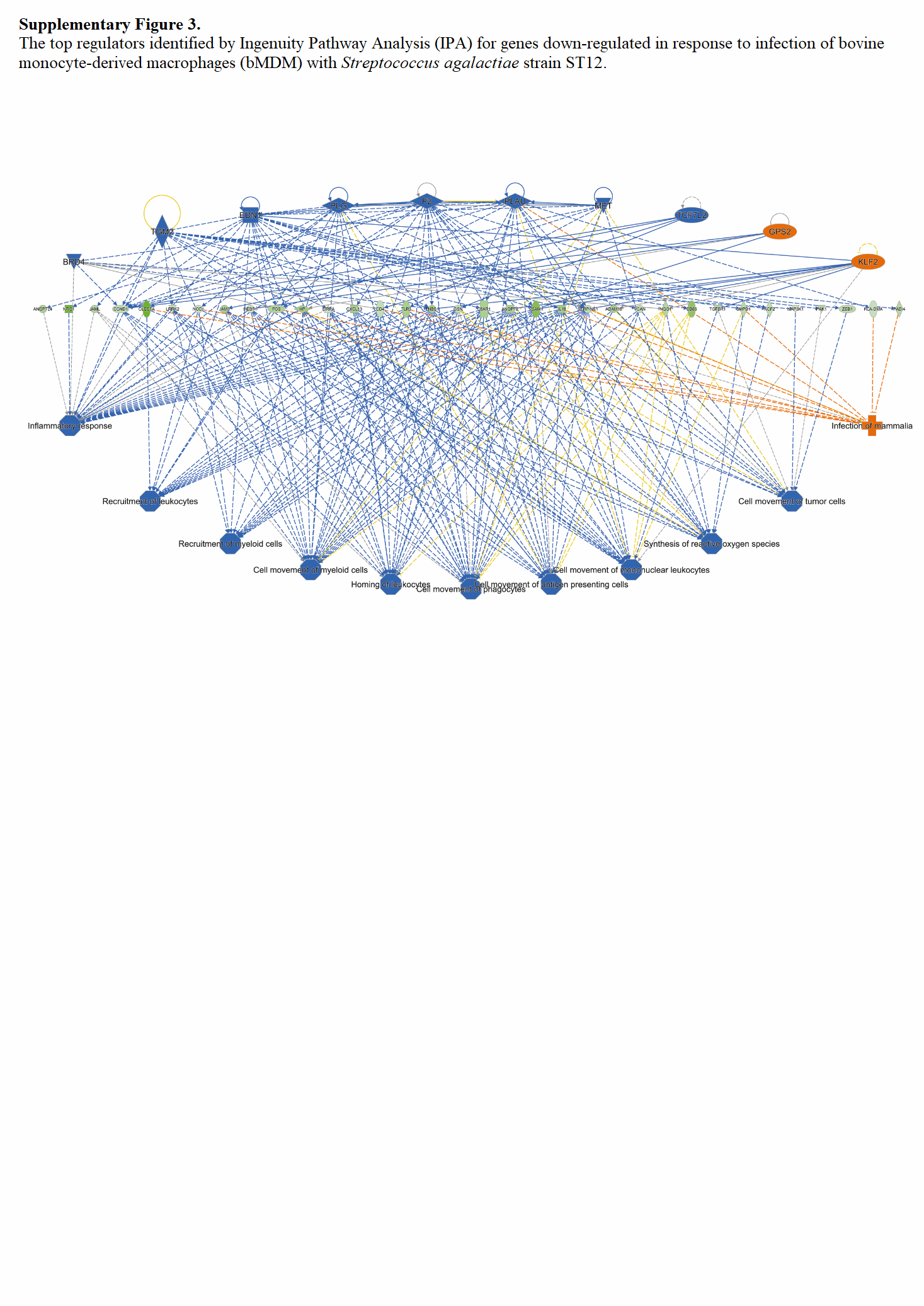

Supplement: Supplementary file 10 [file Image_3.tif]

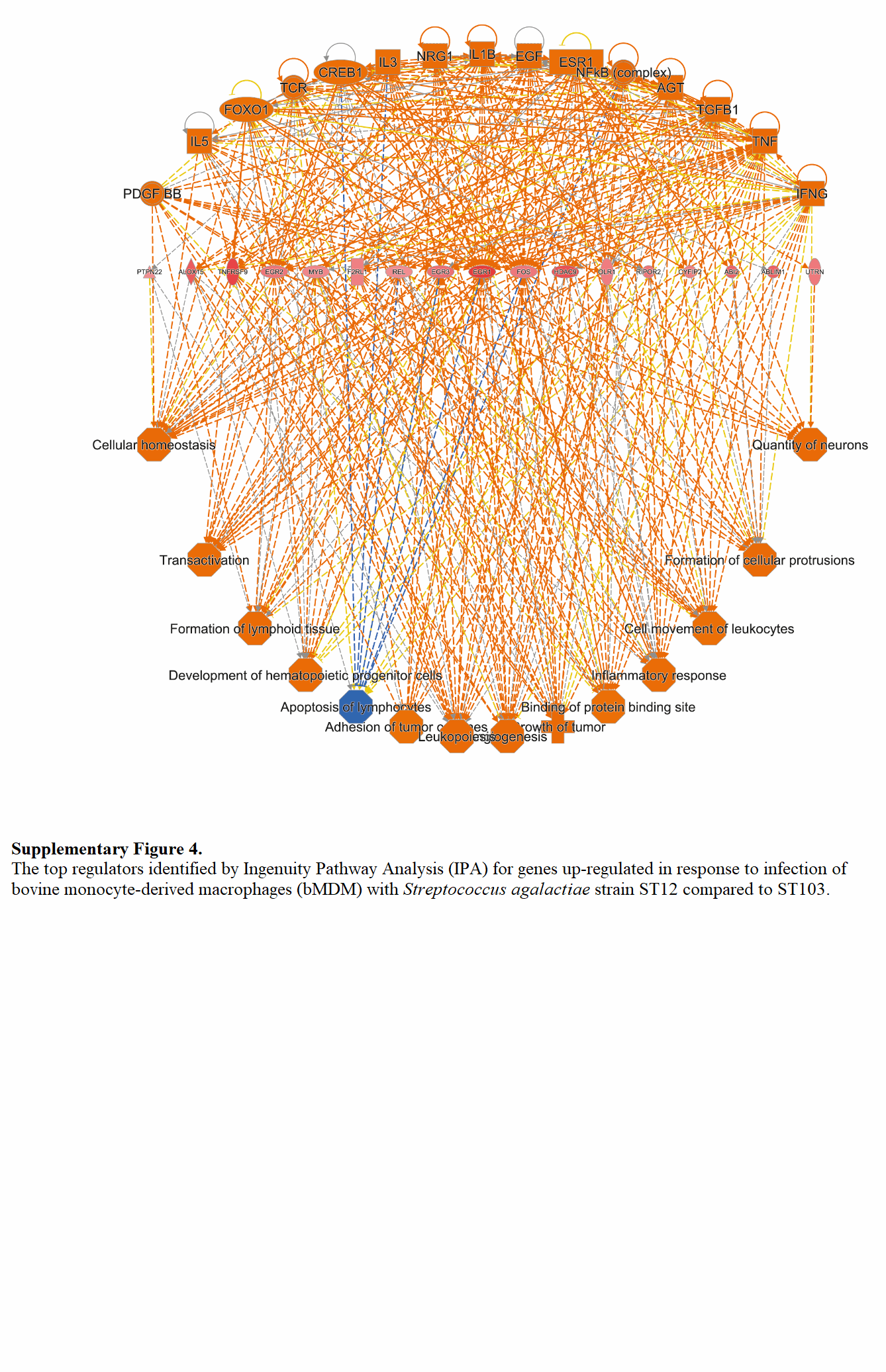

Supplement: Supplementary file 11 [file Image_4.tif]
